# La Trypanosomose Humaine Africaine dans l’espace ivoiro-burkinabé : optimisation des stratégies de surveillance épidémiologique

**DOI:** 10.1051/parasite/2012194389

**Published:** 2012-11-15

**Authors:** R. Kambiré, K. Lingué, F. Courtin, I. Sidibé, D. Kiendrébéogo, K.E. N’gouan, L. blé, D. Kaba, M. Koffi, P. Solano, B. Bucheton, V. Jamonneau

**Affiliations:** 1 Programme National de Lutte contre la Trypanosomiase Humaine Africaine (PNLTHA) au Burkina-Faso 03 BP 7009 Ouagadougou Burkina Faso; 2 Programme National d’Élimination de la Trypanosomiase Humaine Africaine (PNETHA) en Côte d’Ivoire 17 BP 934 Abidjan Côte d’Ivoire; 3 Institut de Recherche pour le Développement (IRD), Unité Mixte de Recherche IRD-CIRAD 177 INTERTRYP, CIRDES Bobo-Dioulasso 01 BP 454 Burkina Faso; 4 Centre International de Recherche-Développement sur l’Élevage en zone Subhumide (CIRDES), Unité de recherches sur les bases biologiques de la lutte intégrée 01 BP 454 Bobo-Dioulasso 01 Burkina Faso; 5 Pan African Tsetse and Trypanosomosis Eradication Campaign (PATTEC) 01 BP 1087 Bobo-Dioulasso 01 Burkina Faso; 6 Projet de Recherche Clinique sur les Trypanosomoses (PRCT) BP 1425 Daloa Côte d’Ivoire; 7 Institut Pierre Richet, Unité de Recherche “Trypanosomoses” 04 BP 293 Abidjan 04, Abidjan Côte d’Ivoire; 8 Université d’Abobo-Adjamé, URES de Daloa, Laboratoire de Génétique Moléculaire et Evolution des Maladies Infectieuses Tropicales BP 150 Daloa Côte d’Ivoire

**Keywords:** trypanosomose humaine africaine, *Trypanosoma brucei gambiense*, surveillance épidémiologique, Côte d’Ivoire, Burkina Faso, lutte, human african trypanosomosis, *Trypanosoma brucei gambiense*, epidemiological surveillance, Côte d’Ivoire, Burkina Faso, control

## Abstract

L’objectif de cet article est de décrire les récentes données de surveillance médicale de la Trypanosomose Humaine Africaine (THA) au Burkina Faso et en Côte d’Ivoire afin (i) de dresser un bilan de la situation actuelle de la maladie dans ces deux pays qui entretiennent depuis plus d’un siècle des liens migratoires, économiques et épidémiologiques intimes et (ii) de définir les stratégies à mettre en place dans l’objectif d’une élimination durable. Les résultats de la surveillance active et passive ont montré que les trypanosomés dépistés au Burkina-Faso ces dernières années sont tous des cas importés provenant de Côte d’Ivoire. Cependant, la réintroduction du parasite est effective et le risque d’une reprise de la transmission existe. En Côte d’Ivoire, plusieurs foyers “historiques” toujours endémiques font craindre des phénomènes de réémergence et de propagation. Dans l’objectif d’une élimination durable de la THA dans ces deux pays, les acteurs de la lutte doivent adapter leur système de surveillance en fonction des différents contextes épidémiologiques. Les prévalences actuelles ne justifient plus, excepté des cas particuliers, l’usage systématique et très onéreux du dépistage actif par prospections médicales exhaustives. Elles tendent plutôt à privilégier des systèmes intégrés aux systèmes de santé nationaux et utiliser des méthodes permettant de cibler les zones prioritaires d’intervention à partir notamment d’un échange d’informations épidémiologiques entre les deux pays. Pour accompagner le processus d’élimination durable, les acteurs de la recherche doivent étudier le rôle respectif des réservoirs humain et animal dans le maintien de la transmission, participer au suivi sur le long terme des cas traités et des suspects sérologiques, et évaluer en termes de coût/efficacité les stratégies mises en place par les Programmes Nationaux afin de les optimiser.

## Abstract (en)

The objective of this paper was to describe recent data from Burkina Faso and Côte d’Ivoire on Human African Trypanosomosis medical monitoring in order to (i) update the disease situation in these two countries that have been sharing important migratory, economic and epidemiological links for more than a century and (ii) to define the future strategic plans to achieve the goal of a sustainable control/elimination process. Results of active and passive surveillance indicate that all sleeping sickness patients diagnosed these last years in Burkina Faso were imported cases from Côte d’Ivoire. Nevertheless the re-introduction of the parasite is effective and the risk of a resumption of transmission exists. In Côte d’Ivoire, few cases are still diagnosed in several historical foci and the fear exists that the disease could reemerge in these foci or spread to other areas. In order to achieve a sustainable elimination of sleeping sickness in these two countries, control entities have to adapt their strategy to the different epidemiological contexts. At the exception of specific cases, the current disease prevalence no longer justifies the use of expensive medical surveys by exhaustive screening of the population. New disease control strategies, based on the exchange of epidemiological information between the two countries and integrated to the regular national health systems are required to target priority intervention areas. Follow-up in time of both treated patients and serological suspects that are potential asymptomatic carriers of parasite is also important. In parallel, researchers need to better characterize the respective roles of the human and animal reservoir in the maintenance of transmission and evaluate the different control strategies taken by National Control Programs in term of cost/effectiveness to help optimize them.

## Introduction

La Trypanosomose Humaine Africaine (THA) ou maladie du sommeil est une affection parasitaire due à un protozoaire de l’espèce *Trypanosoma brucei*. La transmission du parasite à l’homme est assurée par un insecte vecteur hématophage du genre *Glossina*, glossine ou mouche tsé-tsé, présente exclusivement en Afrique subsaharienne. En 1998, 40 000 cas de THA ont été rapportés, mais le nombre de personnes infectées était estimé à 300 000, et celui de personnes à risque à 56 millions (WHO, 1998). En 2009, grâce aux efforts de lutte, le nombre de cas rapportés est passé en dessous de 10 000 (9 689) pour la première fois depuis 50 ans, et le nombre réel de cas est estimé à 30 000 par l’OMS (WHO, 2010 ; Simarro et al., 2011). C’est dans ce contexte que l’OMS et l’Union Africaine (UA – PATTEC) viennent de lancer d’importantes campagnes d’élimination de la THA et des glossines à l’échelle du continent (Kabayo, 2002 ; Simarro *et al.*, 2008 ; Simarro *et al.*, 2010). Cependant, la diminution des prévalences risque de provoquer un désintérêt progressif des bailleurs de fonds, comme ce fut le cas dans les années 1960, mettant en péril l’objectif d’élimination durable de la maladie. En effet, si la THA a été déclarée “résiduelle” à cette époque, on a assisté lors des trois décennies qui ont suivi à une réémergence à l’échelle du continent pour retrouver, à la fin des années 1990, une situation parfois comparable à celle observée au début du 20^ème^ siècle (WHO, 1998).

Les liens migratoires entre le Burkina Faso et la Côte d’Ivoire ont historiquement permis la propagation de certaines pathologies, parmi lesquelles la maladie du sommeil (Courtin *et al.*, 2010a). Les données historiques de l’AOF montrent que, sur un total de 45 000 trypanosomés dépistés de 1931 à 1934, plus de 30 000 provenaient du Burkina Faso (Jamot, 1933). La THA sévissait principalement dans le centre et l’ouest du pays. Les campagnes de luttes médicale et entomologique ont alors permis d’endiguer progressivement l’épidémie et le dernier foyer de THA “autochtone” connu du Burkina Faso fut celui de la Boucle du Mouhoun où 87 malades ont été dépistés de la fin des années 1970 au début des années 1980. Parmi eux, 24 provenaient de la Côte d’Ivoire (Lankoande & Ouedele, 1982). Lors des prospections médicales menées ces dernières années dans des zones définies comme particulièrement à risque de réémergence dans le contexte de la crise ivoirienne et du rapatriement massif de burkinabés venant des foyers ivoiriens, aucun cas autochtone de THA n’a pu être identifié (Rouamba *et al.*, 2009 ; Courtin *et al.*, 2010a) et les seuls cas dépistés au Burkina Faso sont des cas importés de Côte d’Ivoire (Millogo *et al.*, 1999 ; Simarro *et al.*, 2010).

En Côte d’Ivoire, les foyers de THA se sont développés à la faveur de la déforestation pour l’exploitation du bois, puis par la mise en place de cultures de rentes (café, cacao et hévéa principalement). C’est ainsi que dans les années 1930, des épidémies sont décrites dans l’ouest, le sud-est et le centre-ouest du pays (Jamot, 1933 ; Domergue-Cloarec, 1986), où une grande partie de la main d’oeuvre employée provenait notamment de régions burkinabé hautement infestées. Là encore, les campagnes de lutte ont permis de contrôler la maladie à l’aube des indépendances, avec seulement quelques cas reportés annuellement (Vaucel *et al.*, 1963). Mais la diminution des efforts de lutte et l’accentuation de la vitesse de déforestation liée à d’importants flux migratoires, notamment en provenance du Burkina Faso, vont participer à la réémergence d’anciens foyers (Daloa, Bouaflé) et à l’émergence de nouveaux foyers (Vavoua, Sinfra, Bonon) principalement dans le centre-ouest, de la fin des années 1960 aux années 1990 (Vaucel *et al.*, 1963 ; Brengues *et al.*, 1969 ; Duvallet *et al.*, 1976 ; Saliou & Challier, 1976 ; Laveissière *et al.*, 2003 ; Solano *et al.*, 2003 ; Courtin *et al.*, 2010a). Une fois de plus, une lutte médicale active, parfois soutenue par des actions de lutte antivectorielle (LAV) (Laveissière *et al.*, 2003), permettra d’endiguer les phénomènes épidémiques et, ces dernières années, seuls quelques dizaines de cas de THA sont dépistés activement ou passivement dans ces foyers tous devenus hypo-endémiques (Dje *et al.*, 2002 ; Kaba *et al.*, 2006).

Dans le but de soutenir durablement les initiatives d’élimination en cours, il est crucial de mettre en place des stratégies de lutte et de surveillance épidémiologique adaptées aux contextes épidémiologiques. Dans cette démarche, il faudra bien sûr tenir compte du rapport coût/efficacité. L’objectif de cet article est de décrire les données de surveillance médicale de la THA au Burkina Faso et en Côte d’Ivoire en 2010 et 2011, et de discuter ces données afin d’optimiser les stratégies à mettre en place dans ces deux pays qui entretiennent depuis plus d’un siècle des liens migratoires, économiques et épidémiologiques intimes.

## Matériels et Méthodes

### Stratégies de surveillance au Burkina Faso et en Côte d’Ivoire en 2010 et 2011

Il faut préciser qu’il existe deux méthodes de surveillance médicale, l’une active qui repose sur des prospections médicales exhaustives menées par des équipes mobiles, l’autre passive qui repose sur la détection des cas se présentant spontanément dans un centre de santé.

Au Burkina Faso, les cas importés de Côte d’Ivoire peuvent potentiellement se répartir sur tout le territoire. L’une des stratégies passives progressivement mise en place ces dernières années par le PNLTHA est de régulièrement sensibiliser et former, à l’échelle nationale, le personnel médical le mieux disposé à diagnostiquer ces cas et à les traiter. Dès qu’un cas est identifié, il faut vérifier si son environnement est propice ou non (présence de glossines dans la zone) à une possible transmission du parasite. Si c’est le cas, une enquête de proximité est menée pour vérifier que les populations partageant les mêmes espaces que le trypanosomé n’ont pas été infectées. L’autre stratégie consiste à mener des prospections médicales dans les zones à risque de réémergence identifiées à partir de plusieurs critères : historique de la maladie, emprise agricole de la zone, réseau hydrographique, présence de glossines, présence de rapatriés (Courtin *et al.*, 2010a). En Côte d’Ivoire, dans le contexte de sortie de crise, la surveillance passive repose principalement sur le Projet de Recherche Clinique sur les Trypanosomoses (PRCT) de Daloa qui est actuellement le seul centre opérationnel de dépistage passif et de traitement de la THA. Deux autres centres “périphériques” ont aussi la capacité de dépister des trypanosomés : le District Sanitaire de Sinfra et le Centre de Santé Urbain de Bonon, zones qui ont subi les deux plus récentes épidémies de THA. C’est aussi principalement dans ces foyers que se focalisent les prospections médicales menées par les équipes du PNETHA et de ses partenaires, l’Institut Pierre Richet (IPR) et le PRCT.

### Diagnostic et traitement de la THA

Dans les deux pays, le schéma de dépistage actif ou passif est le suivant : Card Agglutination Test for Trypanosomosis (CATT, Magnus *et al.*, 1978) sur sang total (CATT sg), CATT sur dilutions successives de plasma (CATT pl) sur tous les sujets positifs au CATT sg avec détermination de la dernière dilution donnant un test positif, examens parasitologiques (examen microscopique du suc ganglionnaire en cas de présence d’adénopathies cervicales, mini-Anion Exchange Centrifugation Technique (mAECT, Büscher *et al.*, 2009)) pour tous les sujets CATT pl dont le test a été positif au moins à la dilution 1⁄4 (CATT pl ≥ 1⁄4). Pour tous les trypanosomés dépistés, un diagnostic de phase est effectué par la cytorachie et la simple centrifugation du liquide céphalo-rachidien (SC LCR). Les sujets en phase 1 (≤ 5 cellules/μl LCR) sont traités à la pentamidine. Au Burkina Faso, les trypanosomés en phase 2 (> 5 cellules/μl LCR) sont traités au DFMO (schéma 14 jours, (WHO, 1998)) dans l’hôpital le plus proche. En Côte d’Ivoire, les sujets en phase 2 qui étaient traités à l’arsobal (schéma court de 10 jour, Schmid *et al.*, 2005) sont maintenant traités au NECT (Simarro *et al.*, 2012) depuis fin 2010 au PRCT de Daloa.

Le test de la trypanolyse (TL) (Jamonneau *et al.*, 2010) effectué en routine par le Centre Collaborateur de l’Organisation Mondiale de la Santé (CC OMS) de l’Institut de Recherche pour le Développement (IRD) basée au Centre International de Recherche-Développement sur l’Élevage en zone Subhumide (CIRDES) de Bobo-Dioulasso (Burkina Faso) est maintenant retenu dans les stratégies des deux pays pour identifier parmi les sujets séropositifs mais négatifs aux tests parasitologiques (SERO), ceux qui doivent être considérés comme potentiellement porteurs de *Trypanosoma brucei gambiense*.

### Questionnaire épidémiologique

Dans chacun des deux pays, un questionnaire est rempli pour chaque trypanosomé traité. Ce questionnaire renseigne sur l’état civil du sujet, ses lieux de vie (résidences, activités, approvisionnement en eau), son état clinique, les résultats des tests de diagnostic et le traitement administré. Pour les cas traités au Burkina Faso en 2010 et 2011, un questionnaire supplémentaire a été posé concernant leur zone de provenance en Côte d’Ivoire.

## Résultats

### Surveillance au Burkina Faso

**Figure 1.**
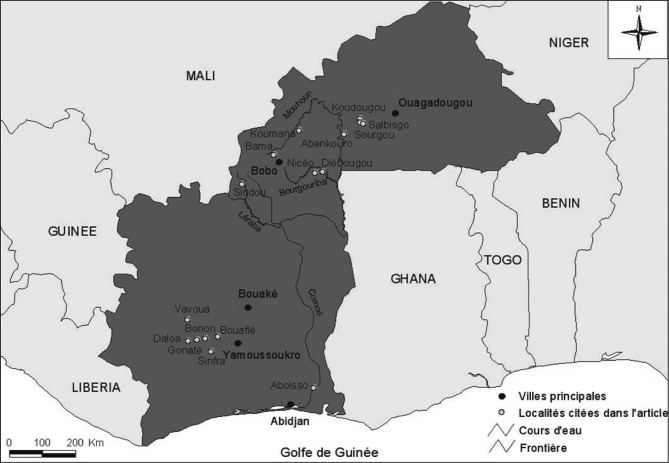
Zone d’étude.

Au total, 4 718 personnes ont été testées au CATT dans le cadre de prospections médicales menées entre 2010 et 2011 (Tableau I) dans les bassins du Mouhoun et de la Léraba (Figure 1). Le CATT pl a été positif (≥ 1⁄4) pour 23 sujets. Les tests parasitologiques (essentiellement la mAECT en absence de ganglions) et la trypanolyse effectués sur ces sujets ont été négatifs.

La surveillance passive a permis de dépister quatre trypanosomés (Tableau II ; Figure 1), trois dans la région de Koudougou (un dans le village de Sourgou et deux dans le village de Salbisgo) et un dans celle de Diébougou (village de Niceo). Ils étaient tous positifs au CATT pl, à au moins un test parasitologique et à la TL. Ces quatre sujets étaient tous résidents dans le centre-ouest ivoirien avant leur dépistage au Burkina Faso (Figure 1). BF1 était élève au collège de Bouaflé, mais passait toutes ses vacances dans les plantations de caféiers et cacaoyers de sa famille dans le village de Sourgou à 28 km de la ville. BF2 et BF3 ont résidé respectivement huit et neuf ans dans la zone de Bonon. BF2 était élève dans le village de Kangreta mais travaillait pendant les vacances dans les plantations familiales de caféiers et cacaoyers. BF3 n’était pas scolarisé et travaillait dans les plantations de cacaoyers de son père dans le campement de Tofla proche du village de Louafla. Et BF4 était cultivateur dans ses propres plantations de cacaoyers dans le village de Gnamanou à côté de la ville de Gonaté.

À part BF2 qui est rentrée au Burkina Faso en 2004 pour finir son cursus scolaire et pour qui les premiers symptômes sont apparus en 2005, les trois autres sont revenus au Burkina Faso pour se faire soigner. C’est au cours de leur itinéraire de soin qu’ils ont été cliniquement suspectés par un infirmier ou un médecin qui connaissait la THA (participation aux formations organisées par le PNLTHA du Burkina Faso). L’examen clinique a montré, en plus de l’existence d’un syndrome infectieux/inflammatoire (fièvres, céphalées, amaigrissement) et de troubles cutanées (prurit, trypanides) qu’ils étaient tous en phase neurologique avancée de la maladie (troubles du sommeil, des réflexes, de la motricité et du comportement). Le PNLTHA a alors dépêché une équipe pour confirmer le diagnostic avec les tests classiques. Malheureusement, BF1 est tombée dans le coma puis est décédée dès le début du traitement. Les trois autres sujets ont été traités au DFMO, BF2 a pu reprendre les cours, BF3 est retourné travailler dans ses plantations en Côte d’Ivoire et BF4 s’est durablement réinstallé dans son village d’origine à Niceo. BF3 a d’ailleurs effectué un contrôle posttraitement au PRCT de Daloa où un cas de rechute a été diagnostiqué (CI12, Tableau III). Il a été traité au NECT.

### Surveillance en Côte d’Ivoire

Au total, 3 071 personnes ont été testées au CATT à Bonon et à Gonaté en 2011 et six sujets ont été positifs au CATT pl (Tableau I). Ces suspects sérologiques ne présentaient pas d’adénopathies cervicales et la mAECT a été négative. Une ponction lombaire a été faite sur deux sujets cliniquement suspects mais la simple centrifugation du LCR a été négative. La trypanolyse a été positive pour trois des six SERO parmi lesquels les deux suspects cliniques. Ces résultats ont été communiqués au PNETHA qui va suivre ces trois sujets potentiellement toujours en contact avec le parasite. Entre 2010 et 2011, 12 trypanosomés ont été dépistés passivement au PRCT de Daloa (Tableau III). Aucun sujet ne présentait d’adénopathies cervicales. Trois sujets négatifs à la mAECT ont été dépistés à la SC-LCR. Tous les sujets étaient en deuxième phase neurologique avec présence de trypanosomes dans le LCR, des cytorachies du LCR comprises entre 60 et 1 198 cellules/μl, et présence de troubles neurologiques (troubles du sommeil, des réflexes, de la motricité et du comportement). Un sujet (CI7) est décédé au premier jour de traitement, et deux cas de rechute post-traitement à l’arsobal (CI2 et CI3) ont été enregistrés lors des contrôles. Un de ces deux sujets a été re-traité au DFMO, l’autre au NECT.

**Tableau. I.** Résultats des prospections médicales menées au Burkina Faso et en Côte d’Ivoire en 2010 et 2011.

			Nombre		
Pays	Localité	Population testée	CATT pl > 14	T+	TL+
BF	Bama	315	7	0	0
BF	Koumana	356	7	0	0
BF	Abenkouro	168	0	0	0
BF	Zone de Sindou	3 879	9	0	0
CI	Bonon	2 511	6	0	3
CI	Gonaté	560	0	0	0

T+ : trypanosomé, TL+ : positif à la trypanolyse, BF : Burkina Faso, CI : Côte d’Ivoire.

**Tableau. II.** Description des quatre trypanosomés dépistés passivement au Burkina Faso.

Identifiant	Sexe	Année de naissance	Résidence au Burkina Faso	Lieu et durée de résidence en Côte d’Ivoire	Profession
BF1	féminin	1993	Koudougou	Bouaflé (17 ans, naissance à 2010)	élève
BF2	féminin	1996	Koudougou	Bonon (8 ans, naissance à 2004)	élève
BF3	masculin	2001	Koudougou	Bonon (9 ans, naissance à 2010)	cultivateur
BF4	masculin	1980	Diébougou	Gonaté (5 ans, 2006 à 2010)	cultivateur

**Tableau. III.** Description des 12 trypanosomés dépistés passivement en Côte d’Ivoire.

Identifiant	Sexe	Année de naissance	Village	Sous-préfecture	Origine	CATT pl	AC	mAECT	SC LCR	Cyto	Traitement
CI1	masculin	1984	Bonon	Bonon	BF	1/32	absence	2	10	274	Arsobal
CI2	masculin	1984	Bonon	Bonon	CI	1/32	absence	3	10	280	Arsobal/DFMO
CI3	masculin	2007	Bonon	Bonon	BF	1/32	absence	40	300	168	Arsobal/NECT
CI4	féminin	1996	Binoufla	Sinfra	BF	1/32	absence	20	6	354	Arsobal
CI5	masculin	1977	Gonaté	Gonaté	BF	1/32	absence	10	20	904	Arsobal
CI6	féminin	1989	Bonon	Bonon	BF	1/32	absence	0	20	1198	NECT
CI7	masculin	1979	Gonaté	Gonaté	CI	1/8	absence	3	10	150	NECT
CI8	féminin	1951	Sinfra	Sinfra	BF	1/32	absence	0	15	60	NECT
CI9	masculin	1997	Sinfra	Sinfra	CI	1/32	absence	20	10	532	NECT
CI10	masculin	1984	Dania	Vavoua	BF	1/8	absence	500	100	120	NECT
CI11	masculin	2007	Biango	Bonon	BF	1/8	absence	100	200	134	NECT
CI12/BF3	masculin	2000	Louafla	Bonon	BF	1/32	absence	0	20	166	NECT

BF : Burkina Faso, CI : Côte d’Ivoire, AC : adénopathie cervicale, Cyto : cytorachie du LCR.

Six trypanosomés provenaient de la zone de Bonon, trois de la zone de Sinfra, deux de la zone de Gonaté et un de la zone de Vavoua (Figure 1). Si tous les sujets sont nés en Côte d’Ivoire, on remarque que neuf d’entre eux sont originaires du Burkina Faso. Malheureusement, le village d’origine n’a pas été renseigné sur les questionnaires et nous n’avons pas non plus de données sur la fréquence de leurs visites dans ces villages d’origine.

## Discussion

Cet article décrit les résultats des activités de surveillance épidémiologique de la THA menées en 2010 et 2011 au Burkina Faso et en Côte d’Ivoire. Les résultats confirment l’absence de cas de THA autochtones au Burkina Faso, mais confirment aussi la réintroduction répétée de *Trypanosoma brucei gambiense* dans le pays. En effet, les quatre trypanosomés dépistés ont très probablement été infectés en Côte d’Ivoire. Ils proviennent du centre-ouest ivoirien qui est une zone endémique de THA, ce qui est confirmé par la répartition des 12 cas dépistés passivement au PRCT de Daloa entre 2010 et 2011.

Parmi les quatre trypanosomés dépistés passivement au Burkina Faso, trois ont été dépistés dans la zone de Koudougou après y avoir séjourné de quelques mois à quelques années. Heureusement, le risque de transmission est très faible dans cette zone où les glossines ont quasiment disparu (Courtin *et al.*, 2010b). Par contre, le trypanosomé de Diébougou a séjourné plusieurs fois et plusieurs mois avant son dépistage dans le village de Niceo (village d’origine) qui est proche d’une rivière (affluent de la Bougouriba, foyer historique de THA, Domergue-Cloarec, 1986) avec une forêt galerie encore bien conservée favorable à *Glossina palpalis gambiensis* où un risque de transmission potentiel existe. Rappelons que les migrations de populations entre le Burkina Faso et la Côte d’Ivoire ont fortement contribué aux dernières épidémies de THA dans ces deux pays. Sur ce point, on remarque que le sujet BF1 qui réside dans le village de Sourgou en Côte d’Ivoire a pour village d’origine Sourgou au Burkina Faso. Sourgou de Côte d’Ivoire est historiquement un village de colonisation de burkinabés venants de Sourgou du Burkina Faso. Ce sujet portant sur les mouvements de populations et la propagation de la THA avec l’exemple très précis des villages de colonisation issus de la région de Koudougou au Burkina Faso fait l’objet d’un article dans ce même volume de *Parasite* (Kiendrébéogo *et al.*, 2012).

Dans le contexte de la crise sociopolitique qu’a vécue la Côte d’Ivoire entre 2000 et 2011 et qui s’est, entre autres, traduite par une augmentation des mouvements de populations entre les deux pays (Courtin *et al.*, 2011), un fort risque de réémergence de la THA a été suspecté dans le sud-ouest (bassin de la Comoé/Léraba) et dans le centre-ouest (boucle du Mouhoun) du Burkina Faso. En effet, de nombreux burkinabés venant des foyers de THA de Côte d’Ivoire se sont installés dans des zones historiques de la maladie où *Glossina palpalis gambiensis* et *Glossina tachinoides* sont encore bien présentes (Rayaisse et al., 2009). Mais toutes les prospections médicales menées dans ces zones n’ont pas identifié de cas de THA (Rouamba *et al.*, 2009 ; Courtin et al., 2010a) et tous les suspects sérologiques identifiés ont été négatifs à la trypanolyse (Jamonneau *et al.*, 2010). Nous confirmons donc les observations de Courtin *et al.* (2010) suggérant que la réintroduction répétée du parasite au Burkina Faso ne semble pas suffisante à une reprise de la transmission en zone de savane pour différentes raisons déjà évoquées dans l’article précité. Cependant, il faut rester prudent et considérer que ces observations sont tirées des prospections médicales qui ne couvrent qu’une infime superficie du territoire burkinabé et qu’une infime portion de la population de ce pays. Il faut aussi tenir compte du temps nécessaire au réveil ou à l’épidémisation de foyers, qui dans le cas d’une maladie chronique comme la THA, peut être de plusieurs années.

Néanmoins, le PNLTHA du Burkina Faso doit tenir compte de ce phénomène dans ses stratégies de surveillance qui doivent d’abord permettre de dépister les cas importés de Côte d’Ivoire. Si les enquêtes menées sur les déplacements de populations entre les deux pays ont montré que les zones d’origine des burkinabés résidant en Côte d’Ivoire concernent principalement le centre et le sud-ouest du Burkina Faso (Courtin *et al.*, 2011), la difficulté réside dans le fait que ces cas importés peuvent potentiellement s’installer dans tout le territoire burkinabé. Il est donc important que le PNLTHA poursuive et renforce ses activités d’information et de formation au diagnostic clinique de la THA auprès du personnel de santé sur toute l’étendue du territoire, en mettant l’accent sur les zones où un risque de réémergence est suspecté.

Le PNLTHA du Burkina Faso doit avoir en permanence une équipe mobile disponible permettant d’aller confirmer dans les plus brefs délais un suspect clinique, puis, en cas de confirmation, le mettre sous traitement et effectuer une enquête épidémiologique de proximité autour du lieu du village du patient, puis enfin, en cas de présence de glossines et d’un risque potentiel de transmission, de mener une enquête diagnostique sur les sujets concernés. L’équipe du PNLTHA à Ouagadougou et celle du CC OMS basée au CIRDES de Bobo- Dioulasso sont opérationnelles à cet effet. Ces équipes mobiles peuvent aussi intervenir en cas de risque accru de réémergence dans une zone donnée (comme ce fut le cas ces dernières années, Courtin et al., 2010a) en menant des prospections médicales exhaustives.

Au lendemain de la grave crise sociopolitique survenue ces dernières années en Côte d’Ivoire, il est difficile d’avoir une idée précise de la situation actuelle de la THA dans ce pays. Les deux dernières épidémies décrites ont successivement touché les foyers de Sinfra avec 600 trypanosomés dépistés entre 1993 et 1997 (Laveissière *et al.*, 2003) et Bonon avec près de 250 trypanosomés dépistés entre 1998 et 2004 (Solano *et al.*, 2003 ; Kaba *et al.*, 2006). Les dernières prospections médicales actives menées dans ces zones pendant la guerre se sont heurtées à des taux très faibles de présentation des populations à risque, reflétant notamment une situation conflictuelle intercommunautaire (Kaba *et al.*, 2006). La baisse apparente des prévalences observées lors des prospections médicales menées depuis le début de la guerre est donc à relativiser, tout comme la baisse progressive du nombre de malades dépistés passivement entre 2004 et 2009 au PRCT de Daloa, qui était probablement en partie due à d’importantes difficultés d’accès aux soins pour ces même populations à risque.

Néanmoins, les résultats de la surveillance passive au PRCT de Daloa semblent confirmer qu’il n’y a pas actuellement de situation épidémique, du moins dans le centre-ouest ivoirien. Cependant, ces mêmes résultats suggèrent que la THA sévit toujours de façon hypoendémique dans cette région et notamment dans les foyers les plus récents comme ceux de Sinfra et Bonon. Ce constat est confirmé par les trois cas dépistés passivement au Burkina Faso provenant de la zone de Bonon et celle de Gonaté entre Bonon et Daloa.

Cette transmission hypo-endémique post-épidémie, encore pourvoyeuse de plusieurs cas annuels à Sinfra et Bonon, semble pouvoir être un phénomène durable. Par exemple, des situations épidémiques ont été enregistrées dans les foyers de Vavoua, Bouaflé et Aboisso dans les années 1970, toutes endiguées par des activités de luttes médicale et/ou antivectorielle (Duvallet *et al.*, 1976 ; Laveissière & Hervouët, 1991). Les dernières prospections médicales menées dans ces foyers dans les années 2000 n’ont pas permis de dépister de trypanosomés. Pourtant, depuis le début des années 1980, quelques trypanosomés (moins de un par an) sont dépistés passivement en provenance de ces zones, comme en témoignent CI10 (résident dans la zone de Vavoua depuis sa naissance en 1984) et BF1 (résidente dans la zone de Bouaflé depuis sa naissance en 1993). Le même phénomène s’observe dans les “anciens” foyers camerounais de Fontem, de Bipindi et de Kribi (Kanmogne *et al.*, 1996). Le maintien de la transmission à long terme peut être du à l’existence d’un réservoir animal de parasites (Njiokou *et al.*, 2006 ; Njiokou *et al.*, 2010) ou à un possible réservoir humain de parasites. Les sujets séropositifs sans confirmation parasitologique (SERO) fréquemment identifiés sur le terrain restent souvent non traités dans les foyers de THA alors qu’il a été récemment prouvé que certains sont des porteurs asymptomatiques (Jamonneau *et al.*, 2010 ; Bucheton *et al.*, 2011 ; Ilboudo *et al.*, 2011 ; Kabore *et al.*, 2011). Des recherches doivent impérativement être menées pour étudier le rôle épidémiologique respectif des réservoirs humain et animal de *T. b. gambiense* dans le maintien de la transmission et/ou dans les phénomènes de réémergence de la THA afin d’optimiser les stratégies de lutte et de surveillance dans l’objectif d’un contrôle durable de la maladie. En attendant, tant qu’un médicament non toxique et facilement administrable ne sera pas disponible, nous suggérons d’effectuer le suivi de ces sujets, sélectionnés sur la base d’un résultat positif à la trypanolyse (TL) qui prouve un contact avec *T. b. gambiense*, tant que le test CATT reste positif (Jamonneau *et al.*, 2010 ; Ilboudo *et al.*, 2011).

Dans un contexte hypo-endémique comme celui actuellement observé dans les foyers du centre-ouest ivoirien, continuer de mener du dépistage actif par des équipes mobiles devient trop onéreux proportionnellement au nombre de cas dépistables. Il est, de plus, maintenant reconnu que cette méthode a tendance à progressivement lasser les populations, surtout quand les prévalences baissent (Robays *et al.*, 2007) et que les prospections sont répétées sur la même population cible. Des réflexions doivent être engagées entre les acteurs de la lutte afin de mettre en place des stratégies mieux adaptées et surtout moins coûteuses en les intégrant, par exemple, en partie ou totalement aux systèmes de santé nationaux. En attendant de disposer de méthodes de diagnostic plus efficaces, évaluées et validées par la recherche, ces stratégies peuvent se baser sur les outils actuellement disponibles comme le CATT sur le terrain ou la TL au laboratoire s’ils sont utilisés à bon escient. Ces réflexions doivent être appuyées par des activités de recherche permettant d’évaluer les stratégies alternatives, notamment en termes de coût/efficacité.

Les résultats présentés et discutés dans cet article (et dans l’article de Kiendrébéogo *et al.*, 2012) ont confirmé le lien épidémiologique important qui existe entre la Côte d’Ivoire et le Burkina Faso en ce qui concerne la THA. Il apparaît évident qu’un partage de l’information entre les acteurs de la lutte dans les deux pays permettrait aussi d’optimiser les stratégies de surveillance. Une enquête sur la provenance en Côte d’Ivoire des trypanosomés dépistés au Burkina Faso permet de cibler des zones à risque de transmission en Côte d’Ivoire. De la même façon, une enquête sur les villages d’origine des burkinabés dépistés en Côte d’Ivoire et notamment sur la fréquence des séjours effectués dans ces villages permettrait de cibler les zones potentiellement susceptibles de favoriser la réintroduction du parasite au Burkina Faso. Chaque programme national devrait notifier tous les cas concernés et renseigner un questionnaire épidémiologique spécifique qui serait à la disposition des deux programmes.
